# The tomato subtilase family includes several cell death-related proteinases with caspase specificity

**DOI:** 10.1038/s41598-018-28769-0

**Published:** 2018-07-12

**Authors:** Sven Reichardt, Dagmar Repper, Alexander I. Tuzhikov, Raisa A. Galiullina, Marc Planas-Marquès, Nina V. Chichkova, Andrey B. Vartapetian, Annick Stintzi, Andreas Schaller

**Affiliations:** 1 Institute of Plant Physiology and Biotechnology, University of Hohenheim, 70593 Stuttgart, Germany; 2 Belozersky Institute of Physico-Chemical Biology, Moscow State University, Moscow, 119991 Russia; 3 Centre for Research in Agricultural Genomics, CSIC-IRTA-UAB-UB, Campus UAB, Bellaterra, Barcelona, 08193 Spain

## Abstract

Phytaspases are Asp-specific subtilisin-like plant proteases that have been likened to animal caspases with respect to their regulatory function in programmed cell death (PCD). We identified twelve putative phytaspase genes in tomato that differed widely in expression level and tissue-specific expression patterns. Most phytaspase genes are tandemly arranged on tomato chromosomes one, four, and eight, and many belong to taxon-specific clades, e.g. the P69 clade in the nightshade family, suggesting that these genes evolved by gene duplication after speciation. Five tomato phytaspases (*Sl*Phyts) were expressed in *N*. *benthamiana* and purified to homogeneity. Substrate specificity was analyzed in a proteomics assay and with a panel of fluorogenic peptide substrates. Similar to animal caspases, *Sl*Phyts recognized an extended sequence motif including Asp at the cleavage site. Clear differences in cleavage site preference were observed implying different substrates *in vivo* and, consequently, different physiological functions. A caspase-like function in PCD was confirmed for five of the seven tested phytaspases. Cell death was triggered by ectopic expression of *Sl*Phyts 2, 3, 4, 5, 6 in tomato leaves by agro-infiltration, as well as in stably transformed transgenic tomato plants. *Sl*Phyts 3, 4, and 5 were found to contribute to cell death under oxidative stress conditions.

## Introduction

Programmed cell death (PCD) is an integral and essential part of the lifecycle of multicellular organisms, in plants as well as in animals. Based on morphological criteria, at least two types of PCD can be distinguished in plants, the so-called ‘vacuolar’ (autolytic) and ‘necrotic’ (non-autolytic) cell death^1,2^. The former is considered to be the predominant type in the course of development, while the latter occurs in response to stress-inducing insults. While the distinction of developmentally-controlled vacuolar cell death and environmentally-induced necrotic cell death is supported by largely distinct transcriptional signatures^3^ there are also examples of plant PCD displaying an intermediate phenotype^2,4^.

PCD in plants exhibits hallmarks of apoptosis in animals including plasma membrane blebbing, cytoplasmic and nuclear shrinkage, chromatin condensation and DNA fragmentation, release of cytochrome C, and the formation of apoptopic bodies. Based on these morphological features plant PCD has been described as ‘apoptopic-like’, but it is still a matter of debate to what extent plant PCD is mechanistically related to apoptosis^4–6^. Plants lack a number of elements critically involved in apoptosis of animal cells including cysteine-dependent aspartate-specific proteases (caspases) as well as B-cell lymphoma-2 (Bcl-2) family proteins^7^. Surprisingly, despite the absence of these core regulators from plant genomes, the ectopic expression of animal pro- and anti-apoptotic Bcl-2 proteins modulates PCD in plants, and the application of specific caspase inhibitors counteracts PCD in various plant models^5,8^. It has thus been suggested that ‘apoptotic like-pathways’ are conserved between plants and animals, and that the conservation of function does not necessarily imply a conservation of primary structure and homology of the proteins involved^6^. Indeed, caspase-like protease activity was frequently shown to be associated with plant PCD, and this activity was attributed to a number of different proteases all unrelated to caspases^4,9^. Plant proteases with caspase-like activities include Vacuolar Processing Enzyme (VPE) and cathepsin B among the cysteine peptidases^10–12^, Aspartyl Protease Cleaving BAG 1 (APCB1) and the β1 subunit of the 26 S proteasome (PBA1) as representatives of the aspartyl and threonine peptidases^13–15^, and saspases, phytaspases and potato subtilase c-3 (*St*SBTc-3) within the clan of subtilisin-like serine peptidases^16–18^.

In contrast to VPE, cathepsin B and PBA1, which also cleave after Asn, Arg and Glu residues, respectively, some proteases within the large subtilase (SBT) family display strict Asp specificity for cleavage of their peptide and protein targets, and hence were named phytaspases^17,19,20^. While Asp in P1 (the position immediately upstream of the scissile bond) is essential, it is not sufficient for substrate recognition by phytaspases. Phytaspase target sites, like those of animal caspases, are marked by an extended stretch of amino acid residues preceding the cleavable Asp bond, thus conferring high selectivity of hydrolysis in both kingdoms. Phytaspase was shown to be involved in PCD in *Nicotiana tabacum* plants, where it mediates cell death responses to biotic (viral infection) and abiotic stresses^17^. Up-regulation of phytaspase levels in transgenic plants markedly enhanced stress-induced PCD, whereas in phytaspase-silenced plants PCD responses were attenuated^17^.

It is therefore likely that the roles played by caspases during apoptosis of animal cells are taken, at least partially, by phytaspases in the course of PCD in plants^9^. However, dictated by structural differences between caspases and phytaspases, their activities are controlled in profoundly distinct ways. Like caspases, phytaspase is synthesized as an inactive precursor protein. Yet unlike caspases, which are controlled largely at the step of proenzyme processing, phytaspase is auto-catalytically and constitutively processed giving rise to the active enzyme that is sequestered in the apoplast (the extracellular matrix of the plant cell)^17^. Sequestration of phytaspase may be regarded as a protective mechanism physically separating the active enzyme from its intracellular substrates, thus avoiding unwanted proteolysis. Upon the induction of PCD, phytaspase re-enters the cell and gets access to its intracellular targets through an unidentified yet apparently specific retrograde transport mechanism^17,21^.

SBTs are represented by large gene families of mostly unknown function in plants, comprising e.g. 56 members in *Arabidopsis thaliana*^22^. Primary structure analysis of SBT precursors has allowed the identification of a single phytaspase gene in *A*. *thaliana*^23^, whereas two different phytaspases have recently been isolated from tomato (*Solanum lycopersicum*) leaves^20^. In analogy to caspases, which include enzymes for the initiation and the execution of apoptosis, a subdivision of labor may be expected if multiple phytaspases exist in a single organism. Therefore, as a first step to elucidate the relevance of phytaspases for different forms of PCD in plants and, possibly, specific functions during different phases of PCD establishment, we set out to identify and characterize the full complement of phytaspases in tomato. 82 SBT genes were identified in tomato. Phylogenetic analysis and comparison to the SBT family in Arabidopsis revealed unequal distribution of individual SBT clades and species-specific patterns of family expansion and tandemly arranged genes, also including tomato phytaspases. Twelve phytaspase genes were identified in tomato that differed widely in expression level and tissue-specific expression patterns. A role in cell death was confirmed for a subset of tomato phytaspases in transient expression assays and in transgenic tomato plants. Analysis of substrate specificity confirmed Asp-specificity for recombinant tomato phytaspases and further indicated that, in analogy with caspases, individual enzymes differ with respect to cleavage preference and thus, by inference, in the repertoire of protein substrates and physiological function.

## Results

### Characterization of the tomato SBT family and identification of phytaspases

**Figure 1:**
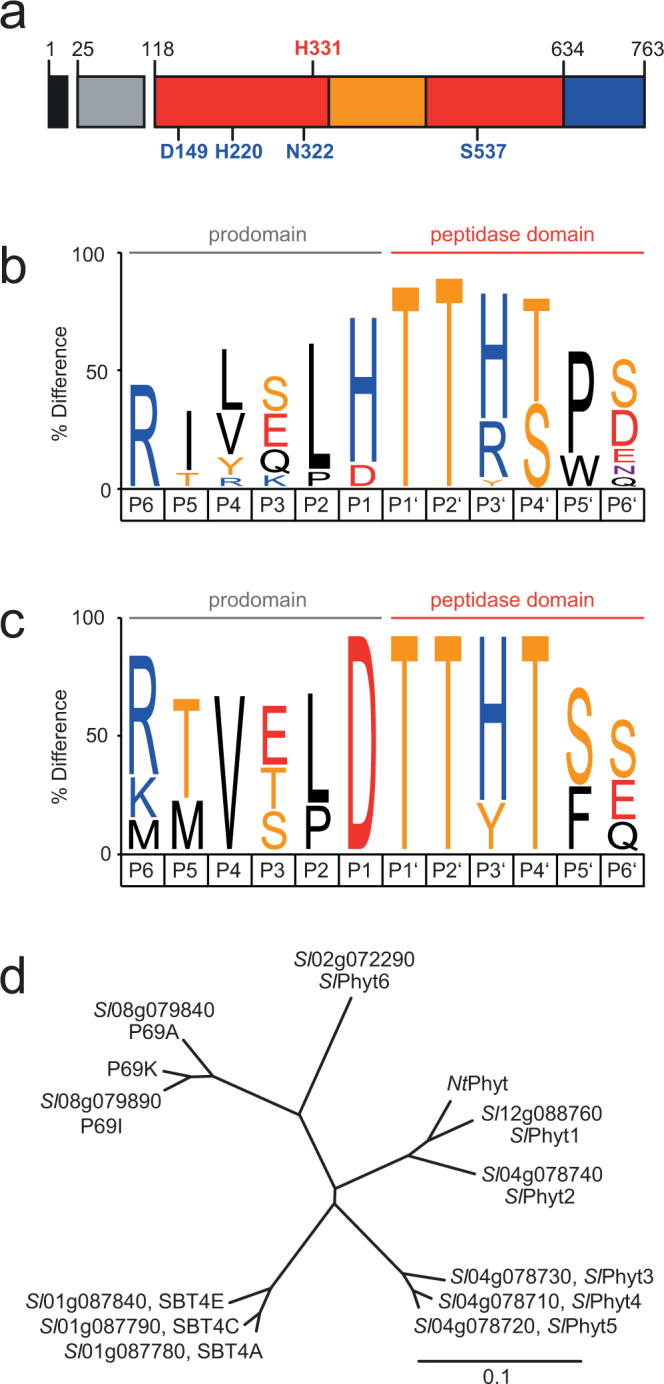
An Asp residue at the prodomain junction characterizes potential phytaspases in tomato. (**a**) Domain structure of plant SBTs. Signal peptide, prodomain, catalytic and protease-associated domains are shown in black, gray, red and orange, respectively. Active site residues are indicated in blue, the diagnostic His residue in red. Numbering of residues according to tobacco phytaspase. (**b**,**c**) Visualization of SBT consensus sequences six positions upstream (P6 to P1) and six positions downstream (P1′ to P6′) of the prodomain junction. IceLogos^57^ were generated for all 82 tomato SBTs (**b**), and for the 12 SBTs with Asp in P1 (**c**). Letter size reflects the relative frequency of an amino acid at this position as compared to prevalence in the tomato (*Solanum lycopersicum*) proteome. Only residues are shown that differ significantly from natural abundance at p < 0.05. (**d**) Neighbor-joining tree of the 12 potential tomato phytaspases compared to *Nt*Phyt from tobacco. The six tomato SBTs comprising His/Lys331 as a second diagnostic residue were named *Sl*Phyt1 to 6. The scale bar represents 0.1 amino acid changes per site.

The BLAST algorithm was used to search for subtilases (SBTs) in the tomato proteome (https://solgenomics.net/; ITAG release 3.1). The retrieved sequenced were curated manually and supplemented with previously published tomato SBTs^20,24–28^. A total of 82 apparently full-length SBT sequences were recovered. They typically share a pre-pro-protein structure comprising an N-terminal signal peptide for targeting to the secretory pathway and a prodomain that serves dual functions as an intra-molecular chaperone and as an inhibitor of the mature enzyme (Fig. 1a)^29^. The prodomain of plant SBTs and of phytaspases in particular is cleaved in an intramolecular autocatalytic reaction^17,30^ and, therefore, the sequence at the prodomain junction is expected to reflect the substrate selectivity of the enzyme. To identify potential phytaspases, tomato SBT sequences were screened for the presence of an Asp residue at the prodomain junction (Fig. 1a). The prevalence of different amino acid residues six positions upstream (P1–P6) and six positions downstream (P1′–P6′) of the scissile bond of all 82 SBTs is shown in Fig. 1b. His is the predominant residue in P1, while two invariant Thr residues in P1′ and P2′ mark the amino terminus of the mature proteases. Twelve tomato SBTs were identified that carry Asp in P1, as the major difference in amino acid composition around the prodomain junction (Fig. 1c). Five of them share a second diagnostic residue, His331 (numbering as in *Nt*Phyt; Fig. 1a), that is located in the S1 substrate binding pocket of the enzyme (the one that accommodates the P1 residue of the substrate). His331 was proposed to bind the P1 Asp and, therefore, to be important for substrate specificity^9^. These five candidates were named *Sl*Phyt1 to 5 (Fig. 1d). Lys replaces His331 in *Sl*Phyt6 (Solyc02g072290), and may also interact with the P1 Asp of the substrate. The remaining six candidates (P69A, P69K, P69I, SBT4A, 4 C, 4E) belong to the previously described P69 and SBT4/3 groups of tomato subtilases^24–26^. Gly is found in the His/Lys331 position of these SBTs. The S1 pocket of these candidates is thus predicted to be wider and less polar. Therefore, it is likely to contribute less to the overall free energy of binding, but it may still be able to accommodate an Asp residue. All twelve candidates and their relation to *Nt*Phyt are shown in Fig. 1d.

**Figure 2:**
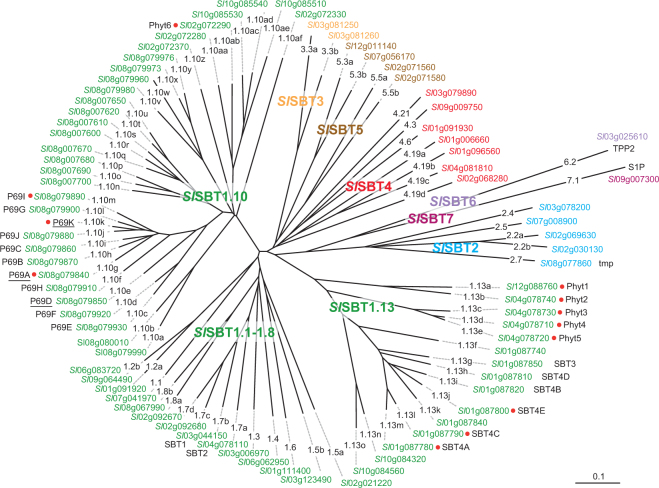
Phylogenetic analysis of the SBT family in tomato. 82 SBT genes were identified in the tomato genome (ITAG release 3.1). The neighbor-joining tree was generated from a sequence alignment of the deduced full-length amino acid sequences in Clustal X^62^ and visualized using GSTree (http://genestudio.com). The scale bar represents 0.1 amino acid changes per site. Different colors are used to distinguish subfamilies *Sl*SBT1 to 7. Different lineages within a subfamily (e.g. lineages 1.1 to 1.13) are named according to Rautengarten *et al*.^31^ and Taylor and Qiu^32^. Small case letters are used to distinguish members within a given lineage (e.g. SBT1.13a to 1.13o). For each SBT, the locus identifier is indicated along with any aliases used in the literature. New unique identifiers are introduced here for the underlined members of the P69 clade that are named ambiguously in the literature (further information in the Supplementary Material). Phytaspase candidates are highlighted by red dots. For simplicity, *Sl* replaces *Solyc* in the gene locus identifiers.

For a closer inspection of the relationship of the 12 potential phytaspases within the tomato SBT family, the 82 sequences were aligned and a phylogentic tree was calculated using the neighbor-joining algorithm in ClustalX. Seven subfamilies were identified corresponding to the early phyolgenetic analysis of Arabidopsis SBTs^31^ and a more recent comprehensive analysis of 2460 SBT sequences form 341 species (Fig. 2)^32^. Subfamilies SBT6 and SBT7 each contain only one member, Tripeptidyl Peptidase 2 (TPP2, Solyc03g025610) and Site-1-Protease (S1P; Solyc09g007300), respectively. These two proteases were initially assigned to subfamily SBT6 in Arabidopsis^31^ that was later shown not to be monophyletic^32^. Following the example of Taylor and Qiu, we therefore place TPP2 and S1P into two distinct subfamilies as *Sl*SBT6.2 (Solyc03g025610) and *Sl*SBT7.1 (Solyc09g007300), respectively. Subfamilies SBT1 to SBT5 show varying degrees of family expansion and they differ substantially between tomato and Arabidopsis (Fig. 2). For example, there are only two genes in tomato subfamily SBT3, as compared to 18 in Arabidopsis, most of which are derived from recent gene duplications in the order of Brassicales (Fig. 2)^32^. Likewise, there are only seven tomato genes in the SBT4 clade, compared to 15 in Arabidopsis (17 according to ref.^32^), most of them in tandem arrays.

Subfamily SBT1, on the other hand, comprises 61 genes in tomato and is thus much larger than the nine-membered subfamily in Arabidopsis. 48 of these genes, most of them tandemly arranged, belong to lineages 1.10 and 1.13 that are not found in Arabidopsis and include many proteases thought to be involved in symbiosis and other biotic interactions (Fig. 2)^32^. All tomato phytaspase candidates are found in these two clades, eight in SBT1.13 and four in SBT1.10. In contrast, only one phytaspase can be identified in Arabidopsis on basis of the diagnostic Asp residue at the prodomain junction (At4g10540)^23^ and this protease belongs to subfamily SBT3 (AtSBT3.8), that is virtually absent from tomato (Fig. 2).

### Cleavage specificity of tomato phytaspases

**Figure 3:**
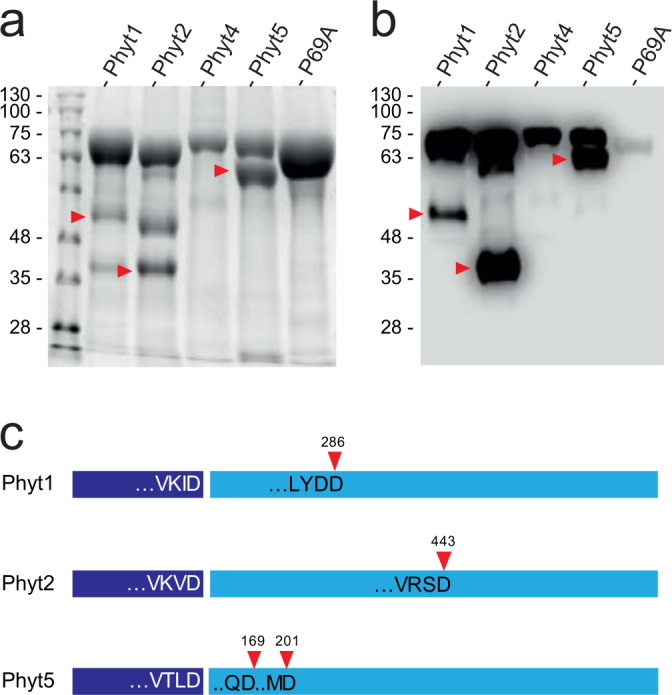
Expression and purification of tomato phytaspases. *Sl*Phyt1, 2, 4, 5, and P69A equipped with a C-terminal His-tag were expressed in *N*. *benthamiana* leaves by agro-infiltration. Five days after infiltration, extracellular proteins were recovered by apoplastic wash. (**a**,**b**) Recombinant phytaspases were purified by metal-chelate affinity chromatography and analyzed by SDS-PAGE and Coomassie-staining (**a**), as well as anti-His immunoblotting and chemiluminescence detection (**b**). Some autolytic fragmentation is observed for Phyt1, 2, and 5. Arrow heads mark the C-terminal fragment. (**c**) Autolytic cleavage sites of *Sl*Phyt1, 2, and 5. The prodomain (dark blue) is cleaved autocatalytically in plant SBTs. The four amino acids upstream of the prodomain junction are shown for *Sl*Phyt1, 2, and 5. Red arrow heads mark the position of autolysis within the catalytic domain (light blue; two possible sites for *Sl*Phyt5) resulting in the fragmentation observed in (**a**) and (**b**).

*Sl*Phyt1 to 6 cDNAs were cloned in order to determine substrate specificity and confirm them as *bona fide* phytaspases. P69A was included in these analyses as one of the candidates lacking His/Lys in the S1 pocket. The seven enzymes, either with a C-terminal His-tag or untagged, were transiently expressed in *Nicotiana benthamiana* by infiltration of Agrobacteria carrying the respective expression constructs on binary T-DNA vectors. *Sl*Phyt3 and *Sl*Phyt6 could not be expressed as His-tagged fusion proteins and, therefore, could not be purified from extracellular (apoplastic) extracts. However, low levels of expression resulted for the untagged proteins, and the activity of these two enzymes was thus assayed in total extracts. His-tagged *Sl*Phyt1, 2, 4, 5 and P69A were expressed at rather high levels and were purified from extracellular extracts to apparent homogeneity by metal-chelate affinity chromatography. However, some autolytic degradation was observed for *Sl*Phyt1, 2 and 5, resulting in two major cleavage products (Fig. 3a). The C-terminal fragment was identified by anti-His immunoblotting (Fig. 3b). In order to identify the internal cleavage sites, the fragments were cut out from the gel, subjected to in-gel tryptic digestion, and analyzed by mass spectrometry. Consistent with the expected Asp specificity of these enzymes, the internal cleavage sites resulting in the major fragments in Fig. 3a,b were identified as D286, D443, and D169 or D201 for *Sl*Phyt1, *Sl*Phyt2, and *Sl*Phyt5, respectively (Fig. 3c). Several additional peptides were observed indicative of further Asp-specific degradation.

**Figure 4:**
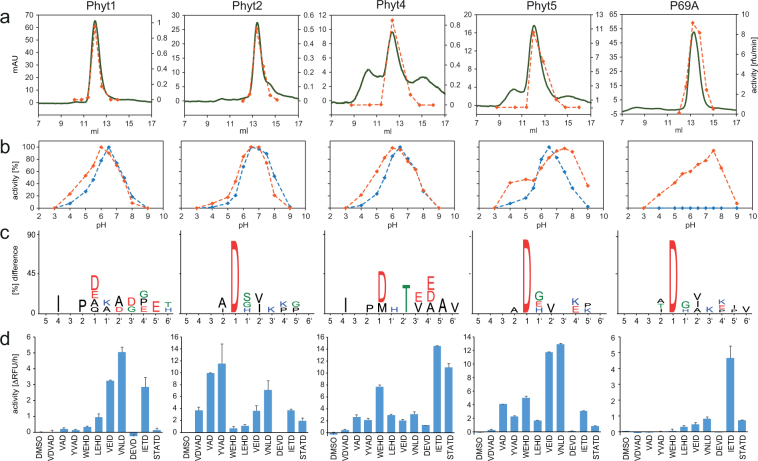
Substrate specificity of tomato phytaspases. (**a**) Size- exclusion chromatography as the final purification step for recombinant phytaspases. Elution was monitored at 280 nm and is shown in m(illi) A(bsorbance)U(nits) in green. The fractions were assayed for VEIDase activity (orange) using a 7-amino-4-trifluoromethyl coumarin (AFC)-peptide-conjugate as fluorogenic substrate at pH 6.5. Activity is shown in arbitrary units as relative fluorescence increase per minute. (**b**) pH optima of recombinant phytaspases were analyzed in a three-component buffer system of constant ionic strength using VEID-AFC (orange) and YVAD-AFC (blue) as fluorogenic peptide substrates. Activity is shown in percent of maximum activity at pH optimum. (**c**) PICS analysis of substrate specificity. IceLogos^57^ show the amino acids that were observed upstream (positions 1–6) and downstream (positions 1′ to 6′) of the scissile bond. Letter size reflects the relative frequency of an amino acid at a given position as compared to natural abundance in the tomato proteome. Only residues are shown that are significantly different from natural abundance at p < 0.05. (**d**) Substrate specificity of tomato phytaspases analyzed with a panel of fluorogenic peptide substrates. The rate of hydrolysis of fluorogenic peptide-AFC conjugates was compared for the different recombinant phytaspases. Substrate peptides comprised two to four variable residues followed by an invariant C-terminal Asp. Activity is shown as relative fluorescence increase per hour. Data represent the mean of two experiments +/− SD.

In order to remove these degradation products and potential further contaminants, the proteins were subjected to size-exclusion chromatography (Fig. 4a), and with as little delay as possible, only the fraction at the peak maximum was used in further analyses. Substrate specificity of the purified enzymes was analyzed by PICS (proteomics identification of cleavage sites)^29,33^. The assay was performed at pH 6.5, which is close to the optimum pH for all enzymes tested (Fig. 4b). A peptide library generated from the Arabidopsis proteome by chymotrypsin digestion was incubated with the test proteases. Cleaved peptides were biotinylated at their newly formed N-termini, isolated on streptavidin beads, and identified by mass spectrometry for cleavage site reconstruction^34^. All phytaspase candidates showed a preference for Asp in P1, which was most pronounced for *Sl*Phyt2, *Sl*Phyt5 and P69A, followed by *Sl*Phyt4 and *Sl*Phyt1 (Fig. 4c). In addition to the pronounced P1 selectivity, we also observed some selectivity in other positions both upstream and downstream of the scissile bond, particularly in P2, P4, P1′ and P2′, and the amino acid preference in these positions differed for the different phytaspase candidates (Fig. 4c).

In order to confirm the PICS results and for further refinement of substrate selectivity, we compared reaction rates with a panel of fluorogenic peptide substrates comprising two to four variable residues and an invariant Asp in P1. While the results of both experiments were generally consistent, the peptide panel revealed differences in specificity that were not immediately apparent from PICS. For *Sl*Phyt1, significant rates were observed only for substrates having Val, Leu, or Ile in P4, with highest activity for VNLD (Fig. 4d), which is consistent with the P4 selectivity for Ile as observed in the PICS assay. For *Sl*Phyt2, a preference for Ala or Ile in P2 was suggested by PICS, and Ala, Leu, and Ile were indeed preferred in peptide substrates. *Sl*Phyt4 showed highest activity for IETD and this is consistent with the PICS result that revealed selectivity for Ile in P4. Another distinguishing feature of *Sl*Phyt4 is the selectivity for Thr in P2′ (Fig. 4c). For *Sl*Phyt5 and P69A, the PICS assay did not indicate much selectivity except for Asp in P1 (Fig. 4c). The peptide panel, on the other hand, revealed considerable differences in substrate specificity for these two proteases: P69A showed highest activity with IETD, while *Sl*Phyt5 was most active with VNLD and VEID substrates. The data show that all the candidates are indeed phytaspases as they share cleavage specificity after Asp in P1. However, the data also indicate clear differences in substrate selectivity depending on the specific residues, particularly in P2 and P4. Differences in substrate specificity were also observed for *Sl*Phyt3 and *Sl*Phyt6 that could not by expressed with an affinity tag for purification, and were thus assayed in crude cell wall extracts of plants expressing the native proteins as compared to empty-vector-infiltrated control plants. *Sl*Phyt3 cleaved YVAD more efficiently than VEID, while neither peptide was a substrate of *Sl*Phyt6 (data not shown). The differences in substrate selectivity observed *in vitro* suggest that tomato phytaspases are likely to cleave different substrates *in vivo*, implying different physiological functions. To verify this notion, we compared the function of tomato phytaspases with respect to their cell death-inducing activity, and analyzed their expression in different tissues of tomato plants.

### Cell death-inducing activity of tomato phytaspases

**Figure 5:**
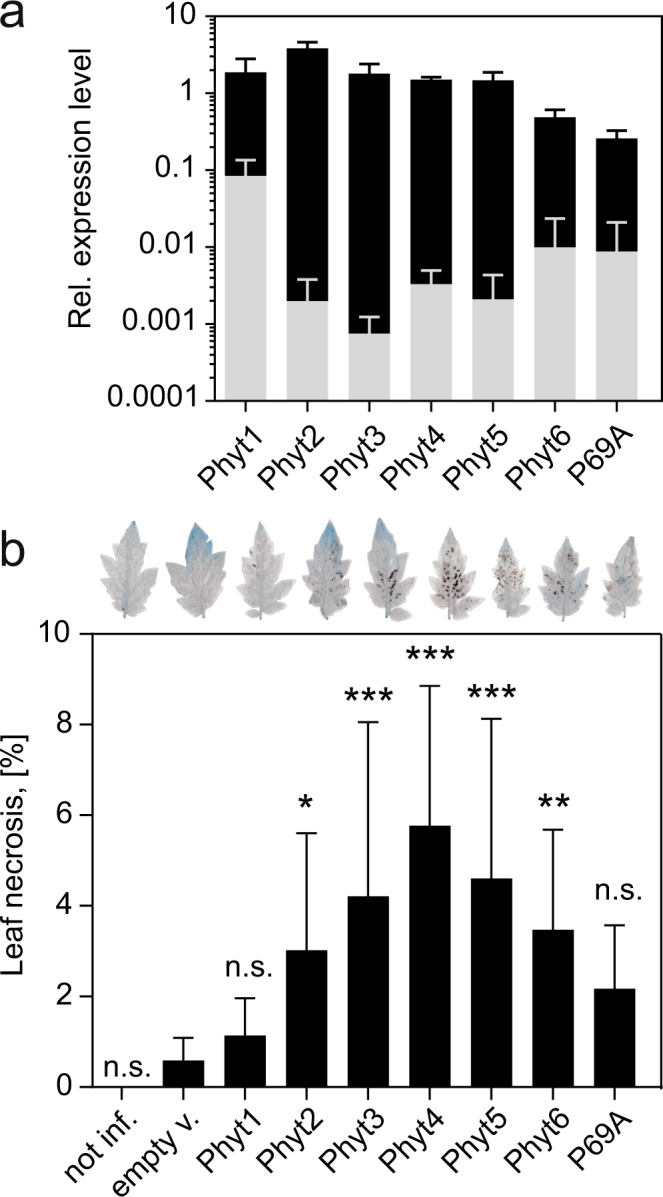
Cell death induction by transient expression of tomato phytaspases in tomato leaves. Three days after infiltration of Agrobacteria for transient expression of tomato phytaspases, cell death was analyzed in tomato leaves by lactophenol/trypan blue staining. (**a**) Over-expression levels in the agro-infiltrated plants (black bars) were analyzed by qRT-PCR and are shown here in comparison to the endogenous transcript level for the different phytaspases in leaves of untreated control plants (gray bars). Elongation Factor 1α was used for normalization. Data represent the mean +/− SD of three independent experiments, each involving the pooled leaf material of at least three infiltrated plants. (**b**) Cell death was quantified as the percentage of necrotic leaf area in phytaspase-expressing leaves as compared to the empty vector (empty v.)-infiltrated and non-treated (not inf.) controls. For each data point, a minimum of 23 leaflets was analyzed from at least nine different infiltrated plants. Error bars represent standard deviation (SD) of the mean. Significant differences to the empty-vector control are shown as *P < 0.05, **P < 0.01 and ***P < 0.0001 (unpaired t-test; n > 23; n.s. = not significant). A representative trypan blue-stained leaflet is shown on top of the bar graph.

**Figure 6:**
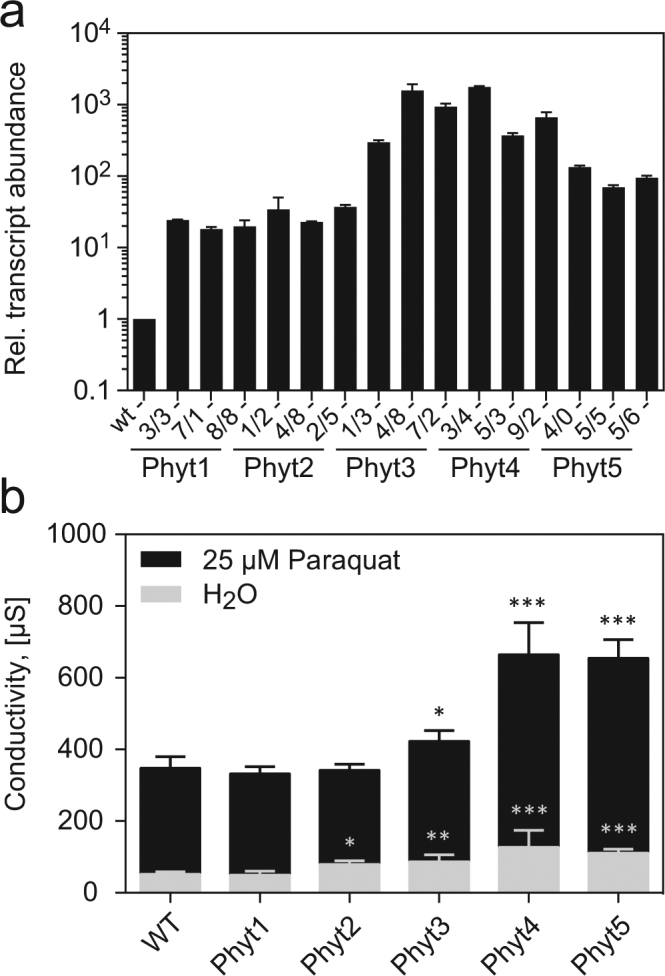
Cell death in transgenic tomato plants expressing tomato phytaspases under control of the CaMV 35S promoter. (**a**) Overexpression levels in transgenic tomato plants. Three independent transgenic lines were analyzed for each phytaspase. Transcript levels were quantified by qRT-PCR and are shown relative to wild-type (WT) levels set at 1. Data points represent the mean for three independent RNA preparations +/− SD. (**b**) Induction of cell death by oxidative stress. Electrolyte leakage (in µS) was analyzed to assess cell death in the wild type (wt) and transgenic lines infiltrated with 25 µM methyl viologen (black bars) compared to water-infiltrated controls (gray bars). Data points represent the mean for 30 leaf discs (10 for each of three independent transgenic lines) +/− standard deviation. Significant differences to wt are shown as *P < 0.01, **P < 0.001 and ***P < 0.0001 (unpaired t-test; n = 30).

*Sl*Phyt1 to 6 and P69A were transiently expressed in tomato leaves by infiltration of agrobacteria carrying the respective expression constructs on binary T-DNA vectors. Overexpression levels ranged from 22-fold when endogenous transcript levels were high (*Sl*Phyt1), to more than 1000-fold for phytaspases that showed very low endogenous expression in leaves (*Sl*Phyt2, 3, 4, and 5; Fig. 5a). Cell death as a result of *Sl*Phyt overexpression was analyzed by trypan blue staining (Fig. 5b). *Sl*Phyt1 or P69A overexpression did not cause any significant increase in cell death. In contrast, increased necrotic leaf area was observed in plants overexpressing *Sl*Phyt2 and 6, and more strongly in response to *Sl*Phyts 3, 4, and 5. These results were confirmed in stable transgenic tomato lines expressing *Sl*Phyt1 to 5 under control of the constitutive Cauliflower Mosaic Virus 35S promoter (Fig. 6a). Cell death was analyzed in leaves of transgenic plants by measuring electrolyte leakage. Consistent with the transient expression assay, increased cell death was observed in plants expressing *Sl*Phyt2, 3, 4, and 5, but not in *Sl*Phyt1 transgenics (Fig. 6b). The apparent inability of *Sl*Phyt1 to induce cell death is in line with its high level of expression in wild-type leaves (Fig. 5a) and it also correlates with its somewhat more relaxed substrate specificity with respect to Asp in P1.

The transgenic plants were then exposed to oxidative stress by treatment with methyl viologen (MV) resulting in the production of reactive oxygen species via the photosynthetic electron transport chain. Hypersensitivity to MV treatment and increased cell death were observed in *Sl*Phyt4 and *Sl*Phyt5 overexpressors and, to a lesser extent, also in plants transformed with *Sl*Phyt3 (Fig. 6b). *Sl*Phyt1 and *Sl*Phyt2 overexpressors showed wild-type levels of MV tolerance (Fig. 6b). Therefore, despite its cell death-inducing activity (Fig. 5b), *Sl*Phyt2 does not seem to contribute to oxidative stress-related cell death. The data indicate that Asp-specific tomato phytaspases are able to trigger cell death when they are ectopically overexpressed in tomato leaves, and that they differ with respect to the specific cell death events they are involved in. *Sl*Phyt1 and P69A do not contribute to cell death, at least not under the conditions analyzed.

### Tissue-specific expression of tomato phytaspases

**Figure 7:**
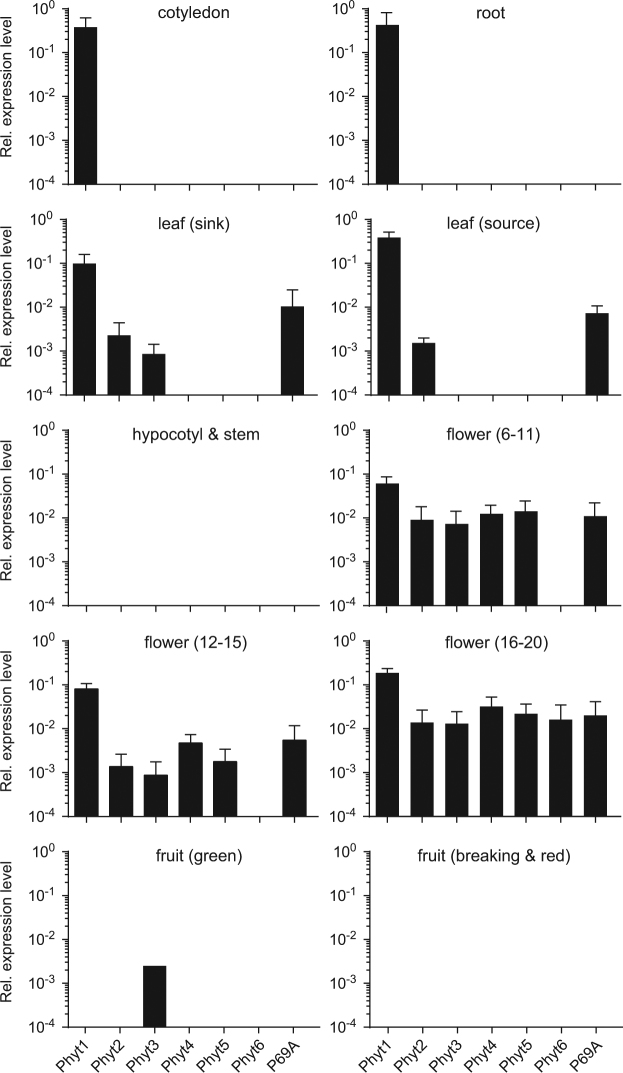
Tissue-specific expression of tomato phytaspases. The levels of phytaspase transcript were analyzed by qRT-PCR in different tissues of tomato plants and normalized to three house-keeping genes (UBI3, Elf1α, bTub). Transcript levels are shown relative to the Elf1α ucontrol. Flower stages (6–11, 12–15, 16–20) as defined by Brukhin and coworkers^63^. Data represent the mean +/− SE of three biological replicates (three independent RNA extractions, each including the pooled tissue of at least four different plants).

To further support the notion of different physiological roles for individual tomato phytaspases we analyzed their expression in different tissues of tomato plants by quantitative PCR. *Sl*Phyt1 showed the highest level and also the most ubiquituous pattern of expression. Its transcript levels were several orders of magnitude higher than those of other phytaspases except in hypocotyls, stems and fruits, where *Sl*Phyt1, like most other phytaspases, was undetectable (Fig. 7). In roots and cotyledons, *Sl*Phyt1 is the only phytaspase expressed. High constitutive expression of *Sl*Phyt1 is consistent with the apparent lack of cell death-inducing activity. *Sl*Phyt2, *Sl*Phyt3 and P69A transcripts were detected at considerably lower levels in flowers and leaves, and *Sl*Phyt3 is the only one found in green fruits. Most phytaspases including P69A were expressed at moderate levels in flowers, and the expression of *Sl*Phyt4, 5, and 6 was detected nowhere else. Phytaspase expression in flowers correlates with many events of cell death during reproductive development, including the degeneration of the tapetum in the course of pollen development, the elimination of three of the four female meiotic products during megasporogenesis, synergid and antipodal cell death of the female gametophyte, and the degeneration of suspensor, nucellus, endosperm, and integuments during embryo and seed development^35^. In contrast to the abundance of phytaspases in flowers, none of them was found in hypocotyls, stems, or fruits in the breaking and red stages (Fig. 7).

## Discussion

Based on the presence of a diagnostic Asp residue at the auto-catalytically cleaved prodomain junction, 12 genes for putative phytaspases were identified among the 82 subtilisin-like proteinases in tomato. With the exception of *SlPhyt1* and 6, most of these genes are clustered on tomato chromosomes one (*SBT4A*, *4 C*, and *4E*), four (*SlPhyt2*, 3, 4, and 5) and eight (*P69A*, *I*, and *K*). They all belong to clades 1.10 and 1.13 of subfamily SBT1 that include many proteases thought to be involved in symbiosis and other biotic interactions and that are not found in Arabidopsis^32^. Such unequal distribution of tandemly arranged genes in taxon-specific clades, e.g. the P69 clade in the nightshade family, suggests that these genes evolved as a result of gene duplication events subsequent to speciation^22,32^. Interestingly, some of these gene duplications must have been relatively recent, because similar observations can also be made in a comparison of two more closely related species, tomato and potato. *SlSBT4*.*19a* (*Sl*1g006660), for example, appears to be orthologous to two tandemly arranged loci in potato (PGSC0003DMP400037004, PGSC0003DMP400037005; Supplementary Fig. S1). Vice versa, the three clustered tomato SBTs P69E, F, and J correspond to a single locus in potato (PGSC0003DMP400028116; Supplementary Fig. S1). Likewise, tomato phytaspases *Sl*Phyt1 and *Sl*Phyt2 are sister to *Nt*Phyt, the only phytaspase characterized in tobacco (Fig. 1d). Based on such observations it has been suggested that the SBT family in different species was shaped by evolutionary forces that are specific for their respective ecological niche, and that we may thus expect many SBTs including tomato phytaspases to be involved in processes related to the interaction of plants with their biotic and abiotic environment^22^. Tandem gene duplication and neo-functionalization has been described as a general pattern for host genes involved in the co-evolution with pathogens or symbionts^32,36–38^ and may have contributed also to the evolution of tomato phytaspases.

Substrate selectivity of five phytaspase candidates was analyzed in a proteomics (PICS) assay revealing strict Asp specificity for *Sl*Phyts 2, 5 and P69A and a somewhat less stringent requirement for Asp in P1 for *Sl*Phyts 1 and 4 (Fig. 4c). *Sl*Phyt1 is the closest homolog of the well-characterized, Asp-specific phytaspase *Nt*Phyt in tobacco^17^. The more relaxed specificity of *Sl*Phyt1 (Fig. 4c) therefore came as a surprise. We cannot formally rule out the possibility that cleavage after residues other than Asp as observed for *Sl*Phyt1 and 4 in the PICS assay is due to some minor impurities. However, since such cleavage was not observed for other *Sl*Phyts and for extracts from empty-vector infiltrated controls that were subjected to the same purification scheme, this explanation seems unlikely. Interestingly, when assayed with a panel of AFC (7-Amino-4-trifluoromethylcoumarin)-coupled fluorogenic peptide substrates, also *Sl*Phyt1 showed strict Asp specificity^20^. It thus seems that Asp specificity of *Sl*Phyt1 may vary with assay conditions, as previously reported for Arabidopsis phytaspase that cleaves specifically after Asp at pH < 6.0, while tolerating several other amino acids in P1 at pH 6.0 to 8.0^23^.

The requirement for Asp in P1 is shared between tomato phytaspases and animal caspases. Other common features include selectivity for the P4 residue, hydrophobic in most cases, and a small uncharged residue (Gly, Ser, Ala) in P1′ (Fig. 4c)^39^. Also similar to caspases in animals, tomato phytaspases showed overlapping specificity and cross-reactivity with commonly used tetrapeptide substrates spanning positions P1 to P4 upstream of the cleavage site (Fig. 4d)^39,40^. Substrate specificity of *Sl*Phyt1 and 5 resembled that of tobacco phytaspase and caspase-6 in mammals^17^ showing high activity with VEID- and VNLD-based peptide substrates. Substrates preferred by caspase-1 (VAD, YVAD) were efficiently cleaved by *Sl*Phyt2, while *Sl*Phyt4 and P69A showed high IETDase activity, similar to caspase-8 (Fig. 4d). Interestingly, the DEVD-based peptide, a typical substrate of the main executioner caspases (caspase-3 and -7)^40^, was not hydrolyzed by any of the tested tomato phytaspases (Fig. 4d).

The differences in selectivity of tomato phytaspases for oligopeptide substrates *in vitro* imply different substrates *in vivo* and, consequently different physiological functions. A caspase-like function in PCD is supported by the findings that five of the seven tested phytaspases (*Sl*Phyts 2, 3, 4, 5, 6) were able to induce cell death when ectopically expressed in tomato leaves, either transiently by agro-infiltration (Fig. 5) or in stably transformed transgenic tomato plants (Fig. 6), and that a subset of those (*Sl*Phyts 3, 4, 5) contributes to cell death under oxidative stress conditions (Fig. 6). A role for these phytaspases in PCD is consistent with several studies showing that inhibition of YVADase (*Sl*Phyt2), TATDase (*Sl*Phyt4), or VEIDase (*Sl*Phyt5) activity by addition of corresponding peptide inhibitors results in cell death suppression in various plant models including tomato cell cultures^8^. While there is strong evidence that caspase-3-like DEVDase activity is required for plant PCD as well^8,41^, none of the tomato phytaspases characterized here, or previously characterized phytaspases from other plant species, showed any appreciable activity toward DEVD-based peptide substrates. It therefore seems likely that this activity is provided either by one of the phytaspases remaining to be characterized or by other unrelated peptidase(s). In fact, two cell death-related proteases with caspase-3-like activity have been characterized recently, the 20 S proteasome β subunit1 (PBA1) and cathepsin B^12,14^. While PBA1 is required for cell death induced by avirulent Pseudomonads and during xylem differentiation^14,42^, cathepsin B mediates cell death during both host and non-host resistance responses^43,44^, oxidative stress^12^, and endoplasmic reticulum (ER) stress^15^.

It becomes clear that in contrast to animals, where Asp-specific proteolysis for PCD regulation is mediated by the different members of the caspase family, plants employ many more proteases from different catalytic types to regulate the different forms of cell death and to provide the different specificities that are required. Such sharing of labor is also observed among the phytaspases in tomato as indicated by differences in substrate specificity of individual enzymes (Fig. 4), their tissue-specific expression patterns (Fig. 7), and their differential contribution to cell death when ectopically expressed or exposed to oxidative stress (Figs 5 and 6).

Their apparent role in PCD does not preclude other or additional physiological functions for plant phytaspases. They belong to the large family of SBTs some of which have recently been shown to be involved in the processing of peptide hormone precursors and the release of the active signaling peptides^22,45^. Interestingly, several plant signaling peptides are flanked by Asp residues within the respective precursor proteins implying Asp-specific hydrolysis for activation. The precursors of systemin^46^, phytosulfokines^47^, CAPEs^48^, and members of the GOLVEN/RGF/CLE-like family of peptides^49^ are thus candidates for processing by phytaspases. Prosystemin, the precursor of the tomato herbivore defense signaling peptide systemin, was in fact shown to be processed by *Sl*Phyt1 and *Sl*Phyt2 *in vitro*^20^. However, given the abundance of phytaspases in tomato, processing by *Sl*Phyt1 and 2 *in vitro* does not necessarily imply that these two proteases also are responsible *in vivo*. The only other known target of phytaspases is the VirD2 protein of *Agrobacterium tumefaciens*^50^, that is important for nuclear import and integration of the bacterial T-DNA into the host plant genome^51^. Phytaspase-mediated cleavage of VirD2 is thus thought to contribute to plant defense against *Agrobacterium* infection^52^. As for prosystemin, the specific phytaspase that is responsible *in vivo* for VirD2 cleavage is still elusive, and so are the physiological substrates of individual phytaspases. The identification of the physiological phytaspase substrates and the matching of substrates with the cognate processing enzymes are important next steps towards a better understanding of phytaspase function in plants, in the maturation of peptide hormones, as well as in the regulation of PCD.

## Methods

### Phylogenetic analysis

To retrieve the full complement of tomato SBT sequences, the BLAST algorithm was used to search for proteins encoded in the tomato genome (https://solgenomics.net/; ITAG release 3.1) and the NCBI databases (http://www.ncbi.nlm.nih.gov). The S8 domain of known tomato SBTs was used as the query. The retrieved sequences were manually curated for misannotated introns and filtered for ‘completeness’ (length between 650 and 800 amino acids, presence of all catalytically important residues of the S8 domain, presence of I9 prodomain). The sequences of previously published SBTs^24–26^ and the orthologues of TPP2 and S1P were added individually. The resulting set of 82 sequences was aligned in ClustalX using the default parameters^53^. The alignment was curated manually on basis of the known SBT domain structure and a phylogentic tree was calculated using the neighbor-joining algorithm in ClustalX. GSTree (http://genestudio.com/gstree) was used to display the tree. For a comparison of tomato and potato SBTs, the amino acid sequences deduced from the 74 *SBT* genes in potato^54^ were trimmed to remove signal peptide and prodomain and aligned with tomato SBT sequences using CulstalX as described.

### Cloning of SBTs for transient expression and tomato transformation

The ORFs of *Sl*Phyt1 (Solyc12g088760), *Sl*Phyt2 (Solyc04g078740), *Sl*Phyt3 (Solyc04g078730), *Sl*Phyt4 (Solyc04g078710), *Sl*Phyt5 (Solyc04g078720), *Sl*Phyt6 (Solyc02g072290), and *Sl*P69A (Solyc08g079840) were amplified by PCR using the primer combinations given in Supplementary Table S1. For the expression constructs to be used in transient expression experiments, six His codons were included in the reverse PCR primer to facilitate purification of the recombinant proteins. PCR-products were cloned into pCR2.1-TOPO (Invitrogen/Thermo Fisher Scientific; Waltham, MA) and amplified in *E*. *coli* DH10B. The identity of all constructs was confirmed by sequence analysis (Macrogen; Amsterdam, The Netherlands). Inserts were mobilized using the restriction sites included in the PCR primers (Supplementary Table S1) and cloned into the respective sites between the CaMV 35S promoter and terminator in pART7^55^. Expression cassettes were then transferred from pART7 into the binary vector pART27 using *NotI*. The vectors were then introduced into *Agrobacterium tumefaciens* strain C58C1 for transient expression in *N*. *benthamiana* or tomato, and into strain GV3101 for stable transformation of tomato plants. Transgenic tomato plants individually overexpressing Phyt1, Phyt2, Phyt3, Phyt4 or Phyt5 (35S:Phyt plants) were generated as described^56^.

### Transient expression in *N*. *benthamiana* and purification of SBTs

*A*. *tumefaciens* C58C1 containing the SBT expression constructs in pART27, or the p19 suppressor of silencing were grown for two days on LB plates with appropriate antibiotics (rifampicin, teracyclin and spectinomycin for C58C1/pART27; kanamycin instead of spectinomycin for C58C1/p19). Bacterial colonies were washed off the plates in 6 ml infiltration buffer (10 mM MES pH 5.6, 10 mM MgCl_2_) and mixed to result in a final OD_600_ of 0.7 for C58C1/pART27 and 1.0 for C58C1/p19. A blunt syringe was used to infiltrate the bacterial suspension into leaves of six-week-old *N*. *benthamiana* plants at the abaxial side. Five days after agro-infiltration, the leaves were harvested and vacuum-infiltrated with 50 mM NaH_2_PO_4_/Na_2_HPO_4_, pH 6.5, 200 mM KCl at 75 mbar. Apoplastic fluid was collected by 7 min centrifugation at 1500 × g and 4 °C. The extract was supplemented with 4 mM imidazole, and subjected to metal chelate affinity chromatography on Ni-NTA agarose following the manufacturer’s (Qiagen; Hilden, Germany) instructions. The eluate was subjected to gel filtration using a Superdex 200 column on a Äkta Purifier chromatography system (GE Healthcare; Freiburg, Germany). Activity was monitored using a fluorogenic caspase substrate containing 7-amino-4-(trifluoromethyl)coumarin (AFC) as the fluorogenic group (Ac-VEID-AFC). Only the fraction at the peak maximum was used for further analysis.

### Substrate specificity of tomato phytaspases

The PICS (proteomics identification of cleavage sites) assay was used for analysis of substrate specificity as described by Schilling and Overall^33,34^ and modified as detailed in ref.^45^ and below. Briefly, a protein extract from Arabidopsis leaves was digested with proteomics-grade trypsin to generate a library of several thousand peptides, that were chemically modified to protect free sulfhydryl and amino groups. The peptide library (200 µg) was digested with the recombinant (His)_6_-tagged phytaspases in 50 mM NaH_2_PO_4_/Na_2_HPO_4_, pH 6.5 containing 200 mM KCl. Phytaspases were added at a protease to library ratio ranging from 1:200 to 1:2000 (w/w). The pH was adjusted to 7.5 by addition of 1 M Na_2_HPO_4_ to facilitate biotinylation of newly generated N-termini. Biotinylated peptides were purified over streptavidin Sepharose (GE Healthcare) and subjected to LC-MS/MS for sequence analysis. Cleavage sites were reconstructed using WebPICS (http://clipserve.dentistry.ubc.ca/pics/)^34^ and cleavage site preference is depicted as iceLogos^57^. Substrate specificity was further analyzed using a panel of fluorogenic peptide substrates at a concentration of 20 µM. Assays were performed at pH 6.5 in presence of 0.5 M NaCl at 30 °C for a total of 3 hours, and relative fluorescence increase was monitored using a FLUOstar OPTIMA reader (BMG Labtech/Helicon; Moscow, Russia) equipped with 405 nm excitation and 520 nm emission filters.

### Analysis of cell death in tomato plants after transient expression of *Sl*Phyts

Tomato (*S*. *lycopersicum* cv. UC82b) plants were grown in the greenhouse at a 16-h photoperiod with supplemental light and a 26 °C/18 °C day/night temperature regime. Plants were fertilized at weekly intervals (GABI plus 12-8-11; N, P, K fertilizer at 2 ml/l). Experimental plants were three weeks old and had 4 to 5 fully developed leaves. A large desiccator was used for vacuum (75 mbar) infiltration of agrobacteria (C58C1) carrying the expression constructs for *Sl*Phyt1, *Sl*Phyt2, *Sl*Phyt3, *Sl*Phyt4, *Sl*Phyt5, *Sl*Phyt6, *Sl*P69A, or the empty vector (pART27) as a control. Bacterial suspensions were infiltrated at an OD_600_ of 0.3, and eight plants were used for each of the expression constructs. Three days after agro-infiltration, the three terminal leaflets of the two oldest leaves were harvested and subjected to lactophenol trypan blue staining to visualize cell death^58,59^. Leaves were scanned at 600 dpi and cell death was quantified as the percentage of dark pixels corresponding to necrotic leaf area. The level of *Sl*Phyt expression was analyzed by qPCR in leaf samples of each of the infiltrated plants.

### Analysis of cell death in transgenic tomato plants expressing *Sl*Phyts

Electrolyte leakage was analyzed to quantify cell death in three weeks old transgenic tomato plants expressing *Sl*Phyts 1 to 5 under control of the constitutive CaMV 35S promoter. For each of the genotypes 36 leaf discs (16 mm) were prepared (4 leaf discs per plant, three plants each for three independent transgenic lines) rinsed in ddH_2_O and vacuum infiltrated in 2.5 ml 25 µM methyl viologen with 1 drop of Tween20 (water/Tween20 as control). Samples were incubated at constant light, 26 °C. Conductivity was analyzed at 0 h, 12 h, 36 h, and 60 h after infiltration using a WinLab Data Line conductivity meter (Windaus-Labortechnik; Clausthal-Zellerfeld, Germany). The level of *Sl*Phyt expression was analyzed by qPCR in leaf samples of each of the transgenic lines.

### Expression analysis by quantitative Reverse Transcriptase (qRT)-PCR

qRT-PCR analysis was performed as described^60^ with minor modifications. Tomato tissues were harvested, shock-frozen in liquid nitrogen and ground to a fine powder. Total RNA was extracted from 100 mg tissue powder of cotyledons, leaves and flowers, 200 mg for roots and fruits, and 300 mg for hypocotyl and stem using peqGOLD Trifast reagent (PEQLAB GmbH; Erlangen, Germany) according to the manufacturer’s instructions. RNA integrity was confirmed by agarose gel electrophoresis^61^. Random hexamers were used as primers for first-strand cDNA synthesis with 2 µg total RNA as the template. Tomato elongation factor 1α, ubiquitin3 and β-tubulin were used as internal reference genes. Primer sequences are given in Supplementary Table S2. Primers were tested on genomic DNA, the amplification product was sequenced to confirm specificity, and cloned into pCR2.1-TOPO (Invitrogen). The cloned genomic fragments were used to generate calibration curves over 5 orders of magnitude. The optimum primer concentration was determined and the resulting efficiency of amplification was in the range of 85% to 105% for each primer combination. Real time PCR was performed in a BioRad CFX Connect real-time PCR system (BioRad Laboratories; Munich, Germany) using SYBR-Green for detection of double-stranded PCR products. The relative fold change in mRNA levels of target genes normalized against the three reference genes was calculated as described^60^. Three independent samples were analyzed for each tissue, each run in triplicate.

### Statistical analysis

Data were analyzed using GraphPad Prism software (San Diego, CA, USA) and are presented as the mean +/− standard deviation (SD). The number of independent experiments (n biological replicates) is given in the figure legends. Statistical significance between two groups was analyzed using Student’s *t-*test. The level of significance is indicated by asterisks in the respective figure legends; n.s., not significant.

### Data availability

All data generated or analysed during this study are included in this published article (and its Supplementary Information file).

## Electronic supplementary material

Supplementary Material
