# Author Correction: The tomato subtilase family includes several cell death-related proteinases with caspase specificity

**DOI:** 10.1038/s41598-020-62487-w

**Published:** 2020-03-24

**Authors:** Sven Reichardt, Dagmar Repper, Alexander I. Tuzhikov, Raisa A. Galiullina, Marc Planas-Marquès, Nina V. Chichkova, Andrey B. Vartapetian, Annick Stintzi, Andreas Schaller

**Affiliations:** 1 Institute of Plant Physiology and Biotechnology, University of Hohenheim, 70593 Stuttgart, Germany; 2 Belozersky Institute of Physico-Chemical Biology, Moscow State University, Moscow, 119991 Russia; 3 Centre for Research in Agricultural Genomics, CSIC-IRTA-UAB-UB, Campus UAB, Bellaterra, Barcelona, 08193 Spain

Correction to: *Scientific Reports* 10.1038/s41598-018-28769-0, published online 12 July 2018

This Article contains an error in the Methods section under subheading ‘Substrate specificity of tomato phytaspases’ where,

“Briefly, a protein extract from Arabidopsis leaves was digested with proteomics-grade trypsin to generate a library of several thousand peptides, that were chemically modified to protect free sulfhydryl and amino groups.”

should read:

“Briefly, a protein extract from Arabidopsis leaves was digested with proteomics-grade chymotrypsin to generate a library of several thousand peptides, that were chemically modified to protect free sulfhydryl and amino groups.”

In addition, there is an error in the legend of Figure 4 where,

“Letter size reflects the relative frequency of an amino acid at a given position as compared to natural abundance in the tomato proteome.”

should read:

“Letter size reflects the relative frequency of an amino acid at a given position as compared to natural abundance in the Arabidopsis proteome.”

