# Supplementary material for: The tomato subtilase family includes several cell death-related proteinases with caspase specificity
Source: Sci Rep. 2018 Jul 12;8:10531. doi: 10.1038/s41598-018-28769-0 (PMC6043521; doi:10.1038/s41598-018-28769-0)
Supplement: Supplementary file 1 — Supplementary Material [file 41598_2018_28769_MOESM1_ESM.pdf]

The tomato subtilase family includes several cell death-related proteinases with caspase specificity

Sven Reichardt, Dagmar Repper, Alexander I. Tuzhikov, Raisa A. Galiullina, Marc Planas-Marqués, Nina V. Chichkova, Andrey B. Vartapetian, Annick Stintzi and Andreas Schaller

1. Supporting text: Nomenclature of P69 subtilases ………………………… S-2

2. Table S1 ……………………………………………………………………… S-3

3. Table S2 ……………………………………………………………………… S-4

4. Figure S1 …………………………………………………………………….. S-5

5. Supporting references ……………………………………………………… S-6

Supporting text: Nomenclature of P69 subtilases

There is considerable confusion in the literature with respect to the naming of individual members of the P69 clade (cf. Figure 2). In an attempt to resolve these issues, we now propose a new nomenclature for P69 subtilases.

In 1999, we performed a first analysis of the subtilase gene family in tomato (1). Twelve new tomato subtilases were described including four that were closely related to P69A and P69B that had previously been characterized by Tornero *et al* (2,3). Therefore, these four new members of the P69 clade had been named P69C (acc. number AJ005171), P69D (AJ006786), P69E (AJ005172), and P69F (AJ005173). At about the same time Jordá *et al.* described a genomic cluster containing four genes in addition to P69A and B and, consequently, they also named these genes P69C, D, E and F (4,5). Of these four genes, only P69C was identical between the studies of Meichtry *et al.* and Jordá *et al.* and the names for P69D, E, and F were ambiguous.

The sequence described by Meichtry *et al.* as P69D was distinct but very similar in sequence to P69A. It now becomes clear that the differences between these two sequences are due to sequencing errors in the originally reported P69A sequence. These two sequences correspond to the same tomato gene with the identifier Solyc08g079840. We now refer to Solyc08g079840 as P69A and to Solyc08g079850 as P69D. With respect to the naming of P69E and P69F we keep the nomenclature proposed by Jordà and coworkers. Therefore, P69F of Meichtry *et al.* (AJ005173) which is identical to P69D has to be renamed as P69D. P69E of Meichtry *et al.* (AJ005172) is different from any of the other genes and has not been annotated in the tomato genome. This gene is now referred to as P69K. The names for all members of the P69 clade with the corresponding Solyc IDs are shown in Figure 2.

Table S1. Oligonucleotide primers for cloning of expression constructs

name	sequence (5' to 3')	*restriction site*
Phyt1-1f	GGGGTACCATGGCTAACTATATTGCCTTATG	*Kpn*I
Phyt1-2247r-His	CCGGATCCTTAATGGTGATGATGATGGTGCAGAGGTTCCACTCTTATGGTAGT	*Bam*HI
Phyt1-2420r	CCGGATCCTGTCTGAGCTGTCTTGTTTGGA	*Bam*HI
-84-Phyt2f	CCCTCGAGTGGCTTCCCATACCTCTCACTTGT	*Xho*I
Phyt2-2253r-His	CCTCTAGATCAATGGTGATGATGATGGTGGTTATGTCCTCTCAAAGGATCCAC	*Xba*I
Phyt2-2465r	CCGGTACCCAGAGTCTGAAGTAATCTGCAAGT	*Kpn*I
Phyt3-1f	GGCTCGAGATGGAATTAGTTAAAGGAGTGCCT	*Xho*I
Phyt3-2327r	GGTCTAGATTACTCTAGTTCTTTATGTACCTTCTTCT	*Xba*I
Phyt4-1f	GGCTCGAGATGGGATTCTTATTGTTCTTATTGTTCC	*Xho*I
Phyt4-2247r-His	GGTCTAGATTAATGGTGATGATGATGGTGCCAAGTACGGATCATTGGTGCA	*Xba*I
Phyt4-2297r	GGTCTAGAGAACCTTTAAACTCATAGTCTAAAAATGC	*Xba*I
Phyt5-1f	GGCTCGAGATGGGATTCTTTTTGTTCTTATTGTTCC	*Xho*I
Phyt5-2250r-His	GGTCTAGATCAATGGTGATGATGATGGTGTGACCAAGTATGGATCATTGGTG	*Xba*I
Phyt5-2406r	GGTCTAGATTTGTACTGTTAGTTACTTACTTCATATTTGCC	*Xba*I
Phyt6-1f	GGCTCGAGATGGATCTGAAATTGATCAGTTTAATCT	*Xho*I
Phyt6-2193r	GGTCTAGATTACATTTTTAAAATGCTATGCGGAAATA	*Xba*I
P69A-1f	GGGGATCCATGGGATTCTTGAAAATCCTTCTTGT	*Bam*HI
P69A-2244r-His	GGTCTAGATTAATGGTGATGATGATGGTGTTGGATCAACAAAAGTGCAATTGG	*Xba*I

Table S2. Oligonucleotide primers for qPCR

target	locus ID	name	sequence (5' to 3')
*Sl*Phyt1	Solyc12g088760	Phyt1-636f	TACAGCTGCAGGAAGTCGTGTTGA
		Phyt1-809r	TCCACTCCATCCTCAATTGCCTGA
*Sl*Phyt2	Solyc04g078740	Phyt2-2153f	TTGATGGTTCATTGAGCTGGA
		Phyt2-2341r	CAGAGTCTGAAGTAATCTGCAAGT
*Sl*Phyt3	Solyc04g078730	-96-Phyt3f	CAATGCTCCATGCCCTTAGTA
		Phyt3-153r	AGAGTGCCATTGCTCATGACT
*Sl*Phyt4	Solyc04g078710	-183-Phyt4f	GCTTAACAATGCACCATGGCA
		Phyt4-20r	TCGGCATGAATGACTTGTCCA
*Sl*Phyt5	Solyc04g078720	Phyt5-2166f	CGGATCGATAACTTGGGTGG
		Phyt-52305r	TTGCCCTCACAATCTAAAAATGGA
*Sl*Phyt6	Solyc02g072290	Phyt6-848f	ATTCTCTAGCAATTGGCGCA
		Phyt6-1056r	TGGTTGATAGGCAGATTCGC
*Sl*P69A	Solyc08g079840	P69A-2384f	CTGCTTCAATGTTTGTTCACACC
		P69A-2590r	GTCAAGACTCAAAGGATGGGG
*Sl*SBT4A	Solyc01g087780	SBT4A-1325f	GATCCAGCAGTGTTCAGGGT
		SBT4A-1512r	GGCCCTCGTGATGAGTATCC
EF1-alpha	Solyc06g009960<br>Solyc06g009970	elf1a-1272f	AGCCCATGGTTGTTGAGACCTTTG
		elf1a-1461r	TTCGAAACACCAGCATCACACTGC
ubiquitin3	Solyc01g056940	ubi3-230f	CTCTTGCCGACTACAACATCC
		ubi3-451r	AGCACCGCACTCAGCATTA
β-tubulin	Solyc04g081490	b-tub-317f	TAGAGCCTGGTACGATGGATAG
		b-tub-450r	CAACTCAGCGCCTTCAGTATAA

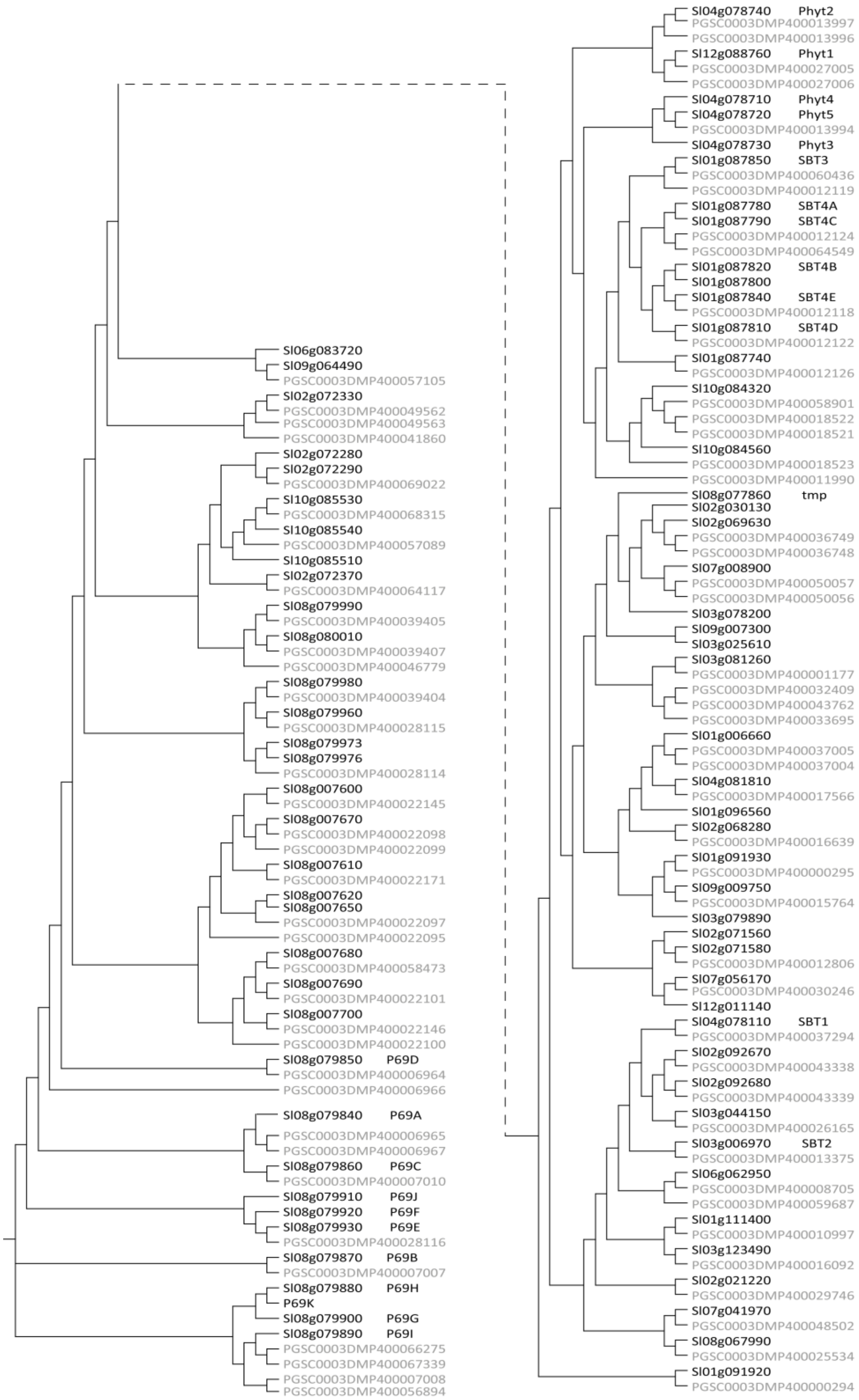

Figure S1. Phylogenetic comparison of tomato and potato SBTs. The 82 tomato SBT sequences were aligned with SBT sequences deduced from the 74 potato genes and a neighbor-joining tree was calculated in ClustalX. Tomato SBTs are shown in black, potato identifiers in gray.

Supporting References

1. Meichtry, J., Amrhein, N., and Schaller, A. (1999) Characterization of the subtilase gene family in tomato (*Lycopersicon esculentum* Mill.). *Plant Mol. Biol.* **39**, 749-760
2. Tornero, P., Conejero, V., and Vera, P. (1996) Primary structure and expression of a pathogen-induced protease (PR-P69) in tomato plants: Similarity of functional domains to subtilisin-like endoproteases. *Proc. Natl. Acad. Sci. USA* **93**, 6332-6337
3. Tornero, P., Conejero, V., and Vera, P. (1997) Identification of a new pathogen-induced member of the subtilisin-like processing protease family from plants. *J. Biol. Chem.* **272**, 14412-14419
4. Jordá, L., Coego, A., Conejero, V., and Vera, P. (1999) A genomic cluster containing four differentially regulated subtilisin-like processing protease genes in tomato plants. *J. Biol. Chem.* **274**, 2360-2365
5. Jordá, L., Conejero, V., and Vera, P. (2000) Characterization of P69E and P69F, two differentially regulated genes encoding new members of the subtilisin-like proteinase family from tomato plants. *Plant Physiol.* **122**, 67-74
